# Analysis of organochlorines and polycyclic aromatic hydrocarbons designed for pollutant biomonitoring in three seabird matrices

**DOI:** 10.1007/s11356-024-34174-0

**Published:** 2024-07-09

**Authors:** Lucie Michel, Bernat Oró-Nolla, Giacomo Dell’Omo, Petra Quillfeldt, Sílvia Lacorte

**Affiliations:** 1 Animal Ecology and Systematics, University of Giessen, Giessen, Germany; 2 Department of Environmental Chemistry, IDAEA-CSIC, Barcelona, Spain; 3 Ornis Italica, Rome, Italy

**Keywords:** POPs, Biomonitoring, Shearwaters, Biological matrices, High-resolution mass spectrometry, Seabirds

## Abstract

Pollutant biomonitoring demands analytical methods to cover a wide range of target compounds, work with minimal sample amounts, and apply least invasive and reproducible sampling procedures. We developed a method to analyse 68 bioaccumulative organic pollutants in three seabird matrices: plasma, liver, and stomach oil, representing different exposure phases. Extraction efficiency was assessed based on recoveries of spiked surrogate samples, then the method was applied to environmental samples collected from Scopoli’s shearwater (*Calonectris diomedea*). Extraction was performed in an ultrasonic bath, purification with Florisil cartridges (5 g, 20 mL), and analysis by GC–Orbitrap–MS. Quality controls at 5 ng yielded satisfactory recoveries (80–120%) although signal intensification was found for some compounds. The method permitted the detection of 28 targeted pollutants in the environmental samples. The mean sum of organic pollutants was 4.25 ± 4.83 ng/g in plasma, 1634 ± 2990 ng/g in liver, and 233 ± 111 ng/g in stomach oil (all wet weight). Pollutant profiles varied among the matrices, although 4,4′-DDE was the dominant compound overall. This method is useful for pollutant biomonitoring in seabirds and discusses the interest of analysing different matrices.

**Supplementary Information:**

The online version contains supplementary material available at 10.1007/s11356-024-34174-0.

## Introduction

Biomonitoring with wild birds is important for observing the current environmental burden and temporal trends of bioaccumulative legacy and emerging contaminants (Becker 2003; Pacyna-Kuchta 2023). Such programs allow for the detection of potential acute and chronic toxic effects and may serve as a basis for the regulation of harmful groups of chemicals (Gómez-Ramírez et al. 2014; Espín et al. 2016; Badry et al. 2022; Kreitsberg et al. 2023). Seabirds can serve as indicators for the health of the marine environment (Parsons et al. 2008) and often have been used to assess persistent organic pollutants (POPs) in ecotoxicological studies (Walker 1992).

Organochlorine pesticides (OCPs) and polychlorinated biphenyls (PCBs), summarised in the following as persistent organic pollutants (POPs), are listed in the Stockholm Convention, a treaty that aims at the elimination or unintentional production of POPs. The greater use of OCPs in agriculture and PCBs for industrial processes led to global pollution of the environment like soil, water, and air and due to their persistence and bioaccumulation potential, ultimately wildlife, as well (Pattnaik et al. 2020). Literature reports a wide range of adverse effects of POPs on birds by interfering with their endocrine, immune, and neural system; reproduction; and development and growth (Hao et al. 2021). Polycyclic aromatic hydrocarbons (PAHs) are formed as by-products of combustion and burning processes of both anthropogenic and natural origin. Certain types of PAHs are subject to monitoring and restrictions by international authorities due to their known carcinogenic and mutagenic properties (Jinadasa et al. 2020). PAHs can be found in air, water, dust, and soil in concentrations that may pose a risk to living organisms. These can enter the body through inhalation, ingestion, and dermal contact. Both POPs and PAHs caused severe toxic effects in seabirds in the past (Burger and Gochfeld 2002). As a consequence of their banning or regulation, long-term studies record decreasing POP and PAH concentrations in seabirds (Rigét et al. 2010, Braune et al. 2007, Bianchini et al. 2022, Elliott et al. 2023). However, due to their persistence and bioaccumulation potential, these compounds are still detected in seabirds worldwide (Costantini et al. 2017; Lewis et al. 2020, 2022; Yamashita et al. 2021), and while legacy POPs are decreasing, total POP concentrations tend to increase due to the accumulation of emerging POPs (Helgason et al. 2008; Jang et al. 2022). Other studies report recent increases in legacy pesticides and stable PCB concentrations in seabirds from the Arctic (Bustnes et al. 2024). Monitoring of legacy POPs continues to be relevant in the light of climate change. Changes in the bioavailability of legacy POPs have been reported due to altered transport and exposure pathways (Braune et al. 2019; Kalia et al. 2021, Corsolini et al. 2022), and increased effective temperatures have been associated with increased OC levels in the blood (Bustnes et al. 2024). Chronic pollutant exposure can affect expression of genes linked to pollutant metabolism and physiological processes (Kreitsberg et al. 2023). It can cause hormone disruption and ultimately lead to behavioural changes that can lower the adaption potential to environmental stochasticity (Esparza et al. 2022) and allocation of parental investment/self-provisioning (Blévin et al. 2020). Exposure of biota to pollution in the environment usually occurs as a complex mixture of different kinds of pollutants (Rochman et al. 2015, Suaria et al. 2016; Gkotsis et al. 2022; Dulsat-Masvidal et al. 2023; Navarro et al. 2023) which in combination can potentially pose threats to seabird populations (Lavers et al. 2014; Hao et al. 2021). Therefore, analytical methods that can cover multiple pollutant classes, targeting both legacy and emerging POPs, are required in biomonitoring studies.

Monitoring pollutants in birds poses challenges to the analytical protocol, as samples of wild birds can be obtained only in limited amounts (Warner et al. 2018). The methods must be highly sensitive and specific while covering a large spectrum of target compounds. A widely used methods for monitoring organochlorine compounds and PAHs are gas chromatography coupled to mass spectrometry (GC–MS). The choice of extraction method depends on the matrix type but usually involves liquid extraction with a non-polar solvent (Moradi et al. 2023). Fatty matrices are sometimes saponified before extraction, but also a solid-phase extraction can be used for the clean-up (JEFCA 2006).

We developed a method based on gas chromatography coupled to Orbitrap mass spectrometry (GC–Orbitrap–MS) to analyse an array of POPs and PAHs in three seabird matrices that represent different exposure stages. Plasma reflects the current body burden, while the liver reflects the accumulated burden of the main detox organ, and stomach oil reflects the uptake by the diet and chick exposure. In this study, we present and evaluate the method’s performance using three tissues and the results of its application to environmental seabird samples. Scopoli’s shearwaters (*Calonectris diomedea*) hold a high trophic level in the marine food chain, exposing them to bioaccumulative contaminants. They have been used as biomonitors for various pollutants in the Mediterranean Sea, such as metals (Renzoni et al. 1986; Ramos et al. 2009); POPs (Roscales et al. 2010); per- and polyfluoroalkyl substances (PFAS) (Escoruela et al. 2018); and PAHs (Roscales et al. 2011). Differences in pollutant concentrations between shearwater populations have been detected, which likely reflect the pollution status of the environment. We used samples of shearwaters breeding on a remote island in the central Mediterranean to test whether our method is sensitive enough to detect targeted bioaccumulative pollutants in samples with expected low to medium concentration levels (Costantini et al. 2017).

In this study, we aimed to facilitate direct comparisons of pollutant concentrations across different exposure stages of a seabird with special interest in the current body burden of rearing adults and fledglings; firstly, the pollutants transferred to the chicks by the stomach oil which is used to feed the chicks. To this end, we employed a single protocol to measure a comprehensive array of target POPs in three seabird matrices.

## Methods

### Environmental sample collection

#### Field site and study species

The fieldwork was conducted in the Scopoli’s shearwater colony of Linosa Island (35° 51′ 33″ N; 12° 51′ 34″ E) located in the Sicily Channel. Between February and October, this small island is the breeding ground of an important population of Scopoli’s shearwaters (Müller et al. 2014; Péron and Grémillet 2013), with 10,000 breeding pairs (Baccetti et al. 2009). Breeding birds lay a single egg per season, and both parents are involved in brood care (Hamer et al. 1987). These long-lived birds (Péron and Grémillet 2013) exhibit a remarkable breeding site and mate fidelity (Mougin et al. 2000). They are top predators foraging exclusively on a marine diet (Zotier et al. 1999; Grémillet et al 2014; Michel et al. 2022), and foraging areas are mainly pelagic, extending along the western part of the Sicily Channel south to the Libyan coast (Cecere et al. 2013, Colominas-Ciurò et al. 2022).

#### Seabird sample collection and preparation

A total of 0.45 mL of whole blood was collected from adult shearwaters (*n* = 11) in July 2020 and from fledglings (*n* = 10) in October 2019 with a syringe by puncturing the tarsal vein. At the station, the samples were centrifuged for 10 min at 2500 rpm, the supernatant plasma was separated, and the samples were stored at − 18 °C until analysis. Liver samples (*n* = 10) were obtained from birds that were killed by a feral dog in May 2021. The carcasses were collected after the incident was discovered and stored at − 18 °C until dissection. In the laboratory, liver samples were freeze-dried and ground in mortars cleaned with acetone and methanol. The water content was determined by weighing before and after the freeze-drying process. The stomach oil of six chicks was sampled in August 2020. A soft plastic tube attached to a 10 mL syringe was gently inserted into the pharynx of a well-fed chick. When the tube reached the end of the oesophagus, 2 mL of stomach oil was extracted by carefully pulling the syringe. Sampled chicks were checked upon on the next days in order to make sure they continued to be well nourished by their parents.

### Pollutant analysis

#### Chemicals and materials used

Analytical standards comprised 24 organochlorine pesticides, 28 polychlorinated biphenyls (PCBs), and 16 polycyclic aromatic hydrocarbons (PAHs). A working solution containing all target compounds was prepared at 0.2 ng/μL in isooctane. We selected six PCB congeners unlikely to be detected in seabird tissues as internal standards (IS) for PCBs and used labelled IS for PAHs and OCPs. Complete compound names, formulae, CAS identifiers, and supplier details are provided in Table S1. Hexane, ethyl acetate, dichloromethane (DCM), acetone, and isooctane were purchased from Merck (Darmstadt, Germany). For the clean-up, Mega Bond Elut–Florisil cartridges (5 g, 20 mL, Agilent Bond Elut) were purchased from Agilent Technologies (Santa Clara, CA, USA).

#### Sample extraction

One hundred microliter plasma, 50 mg liver, and 100 µL stomach oil were spiked with 5 ng of the IS, and 1 mL of hexane-to-dichloromethane (1:1,v/v) was added. The samples were then vortexed for 1 min and sonicated for 10 min in an ultrasonic bath for three consecutive times, followed by centrifugation (3500 rpm; 10 min). The supernatant was cleaned up by passing it through 5-g Florisil cartridges. Conditioning of the cartridges and elution was performed using 10 mL and 24 mL of hexane-to-dichloromethane (1:1), respectively. The extracts were evaporated under a gentle stream of nitrogen to nearly 1 mL using a TurboVap®, transferred to amber chromatographic vials, and further evaporated until near dryness using 400 µL of isooctane as keeper. Samples were stored at − 21 °C until analysis. This extraction condition was compared to three other methods and considered adequate for seabird blood (Oró-Nolla et al. 2024).

#### GC–MS-Orbitrap analysis and data processing

The analysis was performed using a TRACE 130 GC coupled to a Hybrid Quadrupole-Orbitrap™ Mass Spectrometer (GC–Orbitrap–MS) with an HCD (higher energy collision-induced dissociation) and a TriPlus™ RSH Autosampler with a hot split/splitless injector with a single taper liner (78.5 mm × 4 mm ID) from Thermo Fisher Scientific (Waltham, MA, USA) and operated with an electron ionization (EI) source at 70 eV. A volume of 2 μL was injected using a splitless time of 1.5 min at 300 °C. Helium (99.999%) was used as carrier gas at a constant flow rate of 1.0 mL/min. A Phenomenex ZEBRON B-5MS (Torrance, CA, USA) fused silica column of 60 m length × 0.25 mm inner diameter × 0.25 µm film thickness was employed. The oven program was set to 60 °C, held for 3 min, and then increased to 120 °C at 30 °C/min and to 320 °C at 6 °C/min (held for 35 min). The system was calibrated using a perfluorotributylamine calibration solution (FC 43, CAS 311–89-7) to achieve an accuracy of < 0.5 ppm. During the measurement, internal mass calibration was performed using five background ions from the column bleed as lock mass (C3H9Si + , m/z 73.04680; C3H9O2Si2 + , m/z 133.01356; C5H15O3Si3 + , m/z 207.03235; C7H21O4Si4 + , m/z 281.05114; C9H27O5Si5 + , m/z 355.06993) with a mass extraction window of ± 5 ppm. Full scan acquisition was employed at a mass range (m/z) of 70–1000 with a resolving power of 60,000 Full Width at Half Maximum (FWHM), measured at 200 m/z.

The GC–MS data was acquired using Xcalibur 4.4 (Thermo Fischer Scientific), and quantification processing was performed with Trace Finder 5.1 EFS (Thermo Fischer Scientific). Identification was carried out at the specific retention time of each compound and by searching for the exact mass of molecular ions plus specific fragment ions (Table S2) in the chromatogram. The congener identity of PCBs with the same chlorine number was determined by elution order with respect to the IS PCB of the same chlorination level. Quantification was performed using IS calibration and the IS used for each analyte is specified in Table 1. A response factor for each point of the calibration row was calculated. In some quality controls (which are marked with asterisks in Table 2), the signal was re-quantified with external calibration, meaning that we used the uncorrected signal of the sample multiplied by the absolute ng of the spike level (5 ng) divided by the signal of the calibration curve because the signal was very intense. The accuracy of the single mass measurement was described as the mass measurement error (*Δm*_*i*_, in ppm). This was calculated as the difference between the theoretical mass and the experimental mass divided by the theoretical mass and multiplied by 10^6^ as described by Oró et al. (2023).

**Table 1:** Quality parameters for 68 target pollutants. Retention time (in minutes), IS used for correction, main quantification ion (theoretical), mass error of the quantification ion, mean response factor of the calibration curve ± relative standard deviation, *R*^2^, linearity range within the calibration curve (ng/mL), repeatability and reproducibility (both at 5 absolute ng; *n* = 4) and instrumental detection limits (IDL) given in pg injected

Compound	RT (min)	IS	QIon (m/z)	Qion error (ppm)	Response factor ± RSD%	R^2^	Linearity range (ng/mL)	Repeatability (RSD%)	Reproducibility (RSD%)	IDL (pg injected)
Naphthalene	10.54	Naphtalene D8	128.0620	− 0.70	0.94 ± 16.8	0.9985	1–300	0.36	1.30	0.02
Acenaphthene	15.85	Acenaphthene D10	153.0697	− 1.24	0.80 ± 10.2	0.9974	1–120	1.35	10.2	0.02
Acenaphthylene	15.96	Acenaphthene D10	152.0620	− 0.46	0.59 ± 20.9	0.9969	1–300	1.89	7.00	0.00
Fluorene	17.75	Acenaphthene D10	165.0698	− 0.61	0.71 ± 15.1	0.9982	1–120	1.50	3.67	0.04
α-HCH	19.72	Pentachlorobenzene^13^C_6_	180.9373	− 0.17	0.50 ± 17.7	0.9993	1–300	1.89	2.74	0.04
Hexachlorobenzene	19.78	Pentachlorobenzene^13^C_6_	283.8096	− 0.11	0.53 ± 14.9	0.9975	1–200	0.84	1.29	0.03
β-HCH	20.90	Pentachlorobenzene^13^C_6_	180.9373	0.06	0.44 ± 17.9	0.9937	2–300	11.8	22.2	0.07
γ-HCH	21.02	Pentachlorobenzene^13^C_6_	180.9373	< 0.01	0.46 ± 63.0	0.9983	2–300	11.7	15.3	0.03
Phenanthrene	21.43	Phenanthrene D10	178.0777	0.11	0.88 ± 8.3	0.9982	1–120	1.34	1.16	0.01
PCB 24	21.46	PCB 73	255.9607	− 0.47	1.54 ± 10.6	0.9990	2–120	0.66	2.58	0.01
Anthracene	21.63	Phenanthrene D10	178.0777	0.11	0.83 ± 7.4	0.9985	1–120	1.20	9.43	0.01
PCB 16	21.73	PCB 73	255.9607	− 0.31	0.44 ± 12.1	0.9980	2–120	2.78	5.02	0.02
δ-HCH	22.10	Pentachlorobenzene^13^C_6_	180.9373	0.07	0.37 ± 47.9	0.9986	2–300	1.65	4.20	0.01
Heptachlor	23.21	PCB 73	271.8098	− 0.11	0.18 ± 7.1	0.9991	2–120	2.11	8.99	0.05
PCB 52	23.86	PCB 73	291.9190	0.17	1.04 ± 13.8	0.9907	1–120	1.82	2.55	0.02
PCB 49	23.99	PCB 73	291.9190	0.07	0.97 ± 13.2	0.9985	1–120	1.55	2.55	0.03
PCB 38	24.12	PCB 73	255.9613	1.76	1.44 ± 10.1	0.9968	1–120	0.63	2.46	0.01
PCB 62	24.13	PCB 73	291.9190	0.17	1.09 ± 14.8	0.9980	1–120	2.30	7.58	0.02
PCB 65	24.18	PCB 73	291.9190	0.07	1.16 ± 17.0	0.9937	1–120	2.26	5.38	0.02
Aldrin	24.41	PCB 73	262.8560	− 2.05	0.28 ± 11.2	0.9987	1–120	1.41	5.89	0.01
Isodrin	25.40	PCB 73	192.9373	− 0.41	0.24 ± 10.4	0.9970	1–120	2.93	10.0	0.02
PCB 61	25.66	PCB 73	291.9190	0.17	1.30 ± 12.0	0.9968	1–200	1.81	1.46	0.02
Heptachlorepoxide	25.71	PCB 73	352.8436	− 0.34	0.15 ± 6.7	0.9971	2–120	1.55	1.25	0.01
Oxychlordane	25.84	PCB 73	252.8955	− 0.20	0.09 ± 5.9	0.9986	2–120	1.81	3.41	0.03
Fluoranthene	26.14	Phenanthrene D10	202.0777	0.10	0.98 ± 9.2	0.9976	1–120	1.56	5.77	0.06
2,4′-DDE	26.46	4,4′-DDE-D8	245.9998	− 0.12	7.99 ± 11.8	0.9951	1–120	2.60	2.55	0.02
trans-Chlordane	26.49	4,4′-DDE-D8	372.8254	0.05	0.69 ± 13.4	0.9989	2–300	3.56	5.52	0.01
α-Endosulfan	26.49	4,4′-DDE-D8	236.8409	0.38	0.35 ± 19.7	0.9979	2–300	2.70	4.54	< 0.005
cis-Chlordane	26.49	4,4′-DDE-D8	372.8254	− 0.15	0.60 ± 35.0	0.9983	1–300	3.73	17.7	< 0.005
PCB 101	26.64	PCB 97	325.8798	− 0.15	1.57 ± 13.2	0.9986	1–120	2.18	1.18	0.01
PCB 99	26.79	PCB 97	325.8798	− 0.11	1.64 ± 15.8	0.9951	1–200	2.85	3.86	0.01
Pyrene	27.02	Phenanthrene D10	202.0776	− 0.49	1.00 ± 12.7	0.9967	1–120	1.03	6.46	0.01
PCB 116	27.42	PCB 97	325.8804	1.69	1.76 ± 16.0	0.9969	1–200	1.43	2.57	< 0.005
4,4′-DDE	27.52	4,4′-DDE-D8	245.9998	− 0.20	5.16 ± 14.2	0.9982	1–200	2.80	2.35	0.02
PCB 85	27.59	PCB 97	325.8804	1.60	1.02 ± 15.3	0.9975	1–120	2.68	3.20	0.02
PCB 110	27.75	PCB 97	325.8804	1.78	2.12 ± 13.1	0.9933	1–120	1.53	3.51	0.01
Dieldrin	27.77	PCB 151	262.8564	− 0.53	0.24 ± 11.7	0.9983	2–120	3.16	5.58	0.03
2,4′-DDD	27.79	4,4′-DDE-D8	235.0076	− 0.17	7.59 ± 16.2	0.9989	1–300	2.49	3.59	0.01
PCB 77	27.91	PCB 151	291.9190	− 0.14	3.36 ± 11.5	0.9988	1–200	2.29	3.07	0.02
PCB 149	28.45	PCB 151	359.8409	− 0.19	1.60 ± 11.6	0.9971	1–120	1.90	1.01	0.01
Endrin	28.46	PCB 151	262.8566	0.34	0.23 ± 18.4	0.9986	3–300	7.26	33.9	< 0.005
β-Endosulfan	28.85	4,4′-DDE-D8	236.8409	0.38	0.17 ± 18.7	0.9995	3–300	8.93	12.2	< 0.005
2,4′-DDT	28.94	4,4′-DDE-D8	235.0076	− 0.13	5.90 ± 10.6	0.9975	2–300	3.14	19.4	0.02
PCB 146	29.06	PCB 126	289.9032	1.11	0.77 ± 19.9	0.9964	1–200	2.61	8.44	0.01
4,4′-DDD	29.15	4,4′-DDE-D8	235.0077	0.30	2.96 ± 10.8	0.9998	1–300	3.14	19.4	0.02
PCB 153	29.26	PCB 126	359.8410	0.08	0.81 ± 10.3	0.9935	2–200	2.86	9.34	0.01
PCB 118	29.42	PCB 126	325.8804	1.60	1.67 ± 13.1	0.9956	1–120	1.72	5.14	0.01
4,4′-DDT	30.05	4,4′-DDE-D8	235.0077	0.04	0.89 ± 6.6	0.9962	2–120	2.18	33.9	0.01
PCB 138	30.05	PCB 126	359.8410	− 0.17	0.84 ± 13.2	0.9959	1–120	1.95	3.06	0.01
PCB 187	30.50	PCB 126	393.8024	1.32	0.54 ± 15.1	0.9927	2–300	3.50	3.04	< 0.005
PCB 183	30.67	PCB 126	393.8024	1.17	0.60 ± 14.1	0.9960	2–200	1.41	0.79	0.02
PCB 128	30.90	PCB 126	359.8410	− 0.08	0.78 ± 13.1	0.9978	2–120	3.47	3.31	0.01
PCB 167	31.00	PCB 126	359.8410	< 0.01	1.02 ± 16.6	0.9969	2–200	1.51	2.12	0.01
PCB 156	31.67	PCB 157	359.8410	< 0.01	1.33 ± 14.0	0.9975	2–120	0.83	2.03	0.01
Methoxychlor	31.70	PCB 157	227.1068	0.45	0.53 ± 10.3	0.9964	2–120	3.98	24.4	< 0.005
1,2-Benzanthracene	31.83	Chrysene D12	228.0934	− 0.18	0.66 ± 18.3	0.9976	1–300	2.33	9.09	0.01
Chrysene	31.96	Chrysene D12	228.0935	0.13	0.62 ± 18.0	0.9982	1–300	2.30	15.5	0.01
PCB 180	32.10	PCB 157	393.8024	1.32	0.57 ± 17.6	0.9972	2–120	2.58	1.00	0.01
PCB 170	32.94	PCB 200	393.8024	1.17	0.83 ± 7.7	0.9978	2–120	2.02	2.61	0.01
Mirex	33.39	PCB 200	271.8098	− 0.52	0.72 ± 8.0	0.9998	3–120	1.68	3.32	0.01
PCB 189	33.86	PCB 200	393.8024	1.24	0.95 ± 12.7	0.9938	3–120	0.91	8.46	0.03
PCB 194	34.80	PCB 200	429.7605	2.07	0.46 ± 17.4	0.9931	3–120	1.61	8.18	0.04
Benzo[b]fluoranthene	35.86	PCB 200	252.0935	0.04	4.38 ± 11.5	0.9961	1–120	2.93	10.5	0.01
Benzo[k]fluoranthene	35.95	PCB 200	252.0935	0.16	5.54 ± 8.47	0.9995	1–300	4.37	17.7	0.01
Benz[a]pyrene	35.95	PCB 200	252.0935	− 0.04	4.10 ± 4.4	0.9989	2–300	3.91	11.4	< 0.005
Indeno[1,2,3-cd]pyrene	40.68	PCB 200	276.0936	− 0.43	1.44 ± 22.3	0.9972	3–300	2.84	33.6	0.02
Dibenz[a,h]anthracene	40.77	PCB 200	278.1093	0.83	1.17 ± 31.4	0.9976	3–300	7.46	40.2	0.01
Benzo[g,h,i]perylene	41.63	PCB 200	276.0936	0.69	1.57 ± 7.5	0.9973	3–300	3.78	41.8	0.02

**Table 2:** Extraction efficiency assessed on the basis of recoveries in % of the spiked quality controls (5 absolute ng; *n* = 4), method detection limits (MDL) in ng/g w.w. and expanded uncertainty (*U*) for plasma, liver and stomach oil, respectively

	Plasma			Liver			Stomach oil		
Compound	Recovery ± RSD (%)	MDL (ng/g w.w.)	U (%)	Recovery ± RSD (%)	MDL (ng/g w.w.)	U (%)	Recovery ± SD (%)	MDL (ng/g w.w.)	U (%)
Naphthalene	83 ± 5.9	0.43	29	105 ± 8.1	1.60	19	90 ± 10	0.95	33
Acenaphthene	91 ± 5.9	0.79	23	79 ± 13	0.32	17	63 ± 11	0.15	78
Acenaphthylene	81 ± 6.4	1.16	42	87 ± 12	4.67	21	n.d	0.63	133
Fluorene	99 ± 9.4	0.84	25	102 ± 7.6	2.18	16	97 ± 8.3	1.66	22
α-HCH	95 ± 14	1.30	39	90 ± 8.9	5.02	40	83 ± 9.9	3.38	42
Hexachlorobenzene	97 ± 13	1.51	35	84 ± 6.0	3.05	11	93 ± 6.5	2.35	36
β-HCH*	74 ± 15	0.94	70	36 ± 13	4.32	27	25 ± 23	0.33	158
γ-HCH*	94 ± 26	0.94	69	55 ± 23	3.53	35	58 ± 25	3.46	78
Phenanthrene	94 ± 2.9	0.32	15	96 ± 6.2	0.95	12	88 ± 13	0.54	40
PCB 24	87 ± 13	0.32	42	85 ± 11	1.02	15	62 ± 18	0.59	81
Anthracene	99 ± 4.3	0.39	8	95 ± 8.2	1.28	13	83 ± 14	0.75	44
PCB 16	105 ± 3.0	1.13	9	92 ± 13	3.57	15	95 ± 6.1	3.26	12
Heptachlor	98 ± 9.3	0.49	25	n.d	0.16	32	96 ± 5.4	1.10	16
δ-HCH*	135 ± 32	1.52	264	45 ± 18	4.36	37	43 ± 4.0	2.64	121
PCB 52	92 ± 10	1.03	31	80 ± 14	3.47	10	86 ± 6.0	3.14	33
PCB 49	94 ± 12	1.12	34	87 ± 13	3.47	11	92 ± 13	2.48	29
PCB 38	98 ± 10	0.35	28	94 ± 16	1.16	11	97 ± 7.5	1.16	20
PCB 62	95 ± 9.9	0.91	27	83 ± 15	2.64	11	90 ± 11	2.06	35
PCB 65	92 ± 8.3	0.90	26	86 ± 15	2.54	14	93 ± 4.5	2.09	18
Aldrin	86 ± 13	0.26	87	100 ± 8.2	1.07	8	21 ± 62	0.24	230
Isodrin	93 ± 11	0.41	31	83 ± 16	1.40	12	82 ± 10	0.85	44
PCB 61	82 ± 16	0.84	53	89 ± 18	2.66	11	93 ± 12	2.50	33
Heptachlorepoxide	98 ± 9.0	0.49	16	83 ± 8.0	1.23	9	64 ± 14	0.64	65
Oxychlordane	84 ± 9.9	0.45	40	88 ± 15	1.30	20	63 ± 13	0.55	80
Fluoranthene	96 ± 4.5	0.22	14	66 ± 7.4	0.46	13	84 ± 12	0.46	31
2,4′-DDE	95 ± 10	0.45	29	84 ± 5.3	1.28	15	79 ± 2.3	0.83	42
trans-Chlordane	94 ± 5.4	0.49	18	75 ± 11	1.09	19	98 ± 13	1.04	35
α-Endosulfan	100 ± 10	0.53	27	92 ± 14	1.28	30	87 ± 10	0.96	36
PCB 101	94 ± 13	0.61	26	73 ± 9.3	2.57	17	89 ± 13	1.78	40
PCB 99	100 ± 6.7	0.68	18	94 ± 14	3.13	26	85 ± 14	1.93	46
cis-Chlordane	88 ± 15	0.46	46	91 ± 19	1.05	24	90 ± 11	0.71	36
Pyrene	101 ± 7.4	0.22	19	92 ± 5.9	0.72	11	70 ± 11	0.40	60
PCB 116	78 ± 7.1	0.56	46	90 ± 14	1.65	24	82 ± 17	1.23	48
4,4′-DDE	91 ± 10	0.41	32	100 ± 3.0	1.49	12	69 ± 17	0.58	73
PCB 85	90 ± 12	1.41	37	96 ± 8.2	3.39	17	99 ± 8.9	2.94	24
PCB 110	94 ± 9.8	0.63	28	88 ± 14	1.96	15	95 ± 12	1.59	32
Dieldrin	114 ± 5.8	0.61	33	112 ± 15	1.88	32	73 ± 21	0.79	72
2,4′-DDD*	57 ± 8.7	0.32	88	51 ± 8.8	0.78	8	47 ± 14	0.40	116
PCB 77	88 ± 16	0.88	39	75 ± 10	2.31	21	79 ± 14	2.39	55
PCB 149	87 ± 16	1.36	48	100 ± 14	2.06	32	95 ± 12	2.29	22
Endrin	85 ± 14	0.17	46	61 ± 27	0.27	57	53 ± 62	0.13	117
β-Endosulfan*	82 ± 22	0.50	72	43 ± 6.0	0.73	15	9 ± 24	0.07	193
2,4′-DDT	94 ± 5.5	0.44	18	100 ± 7.5	1.59	14	88 ± 3.3	0.93	26
PCB 146	76 ± 16	1.41	61	87 ± 11	2.53	19	100 ± 13	3.21	35
4,4′-DDD	94 ± 12	0.38	34	102 ± 14	1.61	29	97 ± 8.5	1.02	23
PCB 153	92 ± 17	4.10	47	104 ± 7.6	2.56	15	53 ± 10	0.77	98
PCB 118	95 ± 11	0.55	31	99 ± 8.8	1.78	19	94 ± 9.8	2.23	28
4,4′-DDT*	64 ± 14	0.26	107	112 ± 17	1.91	35	25 ± 30	0.29	158
PCB 138	82 ± 12	1.08	45	83 ± 9.9	1.95	19	69 ± 3.6	2.03	63
PCB 187	84 ± 18	1.44	54	111 ± 9.2	6.44	15	98 ± 7.1	3.37	19
PCB 183	87 ± 16	1.56	46	93 ± 18	6.94	13	83 ± 14	3.90	48
PCB 128	75 ± 13	1.32	58	75 ± 7.9	2.22	14	84 ± 6.1	3.14	36
PCB 167	82 ± 16	1.08	45	101 ± 4.9	2.38	16	103 ± 5.7	3.52	31
PCB 156	86 ± 12	1.11	41	112 ± 6.8	2.31	14	98 ± 9.3	3.91	25
Methoxychlor*	74 ± 19	0.42	69	110 ± 10	1.92	23	19 ± 85	0.05	243
1,2-Benzanthracene	99 ± 8.7	0.26	17	93 ± 6.4	1.05	11	63 ± 7.0	0.77	68
Chrysene	102 ± 8.5	0.29	21	86 ± 9.2	1.23	13	88 ± 6.1	1.03	27
PCB 180	99 ± 16	1.96	42	107 ± 16	7.26	15	84 ± 31	3.35	59
PCB 170	93 ± 13	1.72	36	80 ± 14	5.71	18	82 ± 18	3.64	23
Mirex	87 ± 17	0.35	50	88 ± 14	1.74	29	87 ± 14	1.14	44
PCB 189	86 ± 16	1.96	48	94 ± 13	9.38	25	67 ± 22	4.79	83
PCB 194	76 ± 17	1.82	64	93 ± 15	4.41	15	42 ± 61	3.16	191
Benzo[b]fluoranthene	97 ± 5.2	0.24	15	84 ± 12	0.97	30	90 ± 10	2.91	33
Benzo[k]fluoranthene	101 ± 3.7	0.24	10	82 ± 12	0.92	27	83 ± 14	2.89	49
Benz[a]pyrene	97 ± 7.9	0.29	22	85 ± 5.9	0.99	13	77 ± 18	1.07	63
Indeno[1,2,3-cd]pyrene	89 ± 14	0.43	35	81 ± 6.7	4.41	16	83 ± 11	3.04	43
Dibenz[a,h]anthracene	100 ± 11	0.98	30	86 ± 18	3.41	29	68 ± 22	3.10	98
Benzo[g,h,i]perylene	92 ± 10	0.51	31	104 ± 7.8	5.47	18	65 ± 34	2.11	104

^*^Quantified with external calibration using the signal of the calibration curve at 20 ng/mL, due to signal intensification

#### Quality assurance and quality controls

To determine the recovery of our method, we used spiked samples of human plasma and food-grade chicken liver. As we did not have a comparable matrix, which would be expected to contain low levels of organochlorine contaminants for stomach oil, we used a pooled sample of shearwater stomach oil. Four replicates of each matrix (plasma, liver, stomach oil) were spiked with 5 ng of the analytical standard mixture (Table S1) and 5 ng of the IS and extracted as indicated before. Potential background contamination from laboratory materials or solvents was assessed by analysing procedural blanks. To determine the initial contamination load of the spiked samples, we measured one matrix blank per sample type. We monitored potential sample carryovers or signal fluctuations and drift by performing repeated injections of solvent blanks and standard solutions. Repeatability was calculated as the relative standard deviation of five consecutive replicates of a standard at a concentration of 20 ng/mL was calculated. Instrumental reproducibility was determined by calculating the relative standard deviation of four replicates measured along the sequence. The calibration curve consisted of ten points, with vial concentrations ranging from 1 to 300 ng/mL. The limits of detection and quantification of the instrument (IDL, IQL) were determined as three and ten times the concentration of the lowest calibration point divided by the signal-to-noise ratio of the instrument. The method limits of detection (MDL) for each matrix were determined as three times the measured concentration of a spiked sample divided by the signal-to-noise ratio of the instrument. Matrix effects (MEs) were calculated for the three matrices to evaluate the method performance. MEs greater than 20% are considered weak, between 20 and 50% medium and those greater than or equal to 50% have strong effects (Li et al. 2016). The performance of the analytical procedure was evaluated by calculating the method uncertainty (U) for the three matrices as reported in Oró-Nolla et al. (2023). U is expressed as a percentage, and values below 50% indicate satisfactory robustness and reliability of the method. The mean recovery percentages of the spiked samples and MEs as well as MDLs in ng/g (w.w.) are presented based on the four repetitions for each matrix. The results are reported in ng/g wet weight (w.w.) as plasma and stomach oil were extracted as liquids and liver a freeze-dried powder. The mean moisture content of livers was determined as 32.5 ± 1.3% by weighing the sample before and after the freeze-drying process and later converted to wet weight using this factor.

## Results and discussion

### Quality parameters and recoveries

Table 1 shows the instrumental and methodological quality parameters. Mass errors fluctuated negatively and positively around the mean of 0.18 ± 0.75 ppm with only aldrin and PCB 194 exhibiting a higher mass error of − 2.05/2.07 ppm, respectively. The linearity of the ten-point calibration curve, which covered concentrations between 1 and 300 ng/mL, was not always obtained in the higher calibration points. To achieve *R*^2^ values greater than 0.99 and a low relative standard deviation of the mean response factor for all analytes, we excluded some of the higher calibration concentrations. As a result, the linearity range varies from compound to compound but still covers two orders of magnitude, as presented in Table 1. The relative standard deviation of the mean response factor was well below 20% (mean 13.1 ± 3.99%) for all compounds except for two HCH derivates (δ-, γ- HCH), cis-chlordane, and dibenz[a,h]anthracene. The repeatability of the method was well below 10% (mean 2.44 ± 1.49%) for all compounds except for β-HCH and γ-HCH, which was 12%. Reproducibility for most compounds was well below 20% (mean 6.23 ± 5.21%). Exceptions were observed for 3 PAHs (indeno[1,2,3-cd]pyrene, dibenz[a,h]anthracene, benzo[g,h,i]perylene), OCPs (endrin, 4,4′-DDT, methoxychlor), which showed some variations along the sequence. IDL are reported in absolute pg injected (injection volume 2 µL) and ranged between < 0.005 and 0.07.

All 68 targeted compounds were recovered in the spiked plasma, while acenaphthylene was not detectable in stomach oil, and heptachlor was not detectable in the liver. Sixty-five out of 68 targeted compounds in human plasma and 60 in chicken liver were within the recovery range of 80–120%. In pooled shearwater stomach oil, 56 compounds were in the range of 60–120%. The extraction RSD was > 20% in two compounds in plasma (δ-, and γ-HCH) and liver (γ-HCH and endrin) and in ten compounds in oil (ß-HCH, γ-HCH, aldrin, ß-endosulfan, endrin, 4,4′-DDT, methoxychlor, PCB 180, PCB 194 and benzo[g,h,i]perylene). We found signal intensification in plasma for seven OCPs (ß-HCH, δ-HCH, γ-HCH, 4,4′-DDT, methoxychlor, 2,4′ DDD, ß-endosulfan), in the liver for three OCPs (4,4′-DDT, methoxychlor, 2,4′-DDD) and stomach oil only for the two DDT metabolites (4,4′-DDT, 2,4′-DDD). In these cases, we re-calculated the extraction efficiency by external calibration to obtain more realistic recoveries. With this re-quantification, only δ-HCH in plasma recovered over 120%. We attribute these variations to matrix effects (Table S3), as we already subtracted potential contamination of the matrix or procedural blanks. Table S4 in the supporting info compares the recoveries with internal and external standard quantification. δ-HCH in plasma showed the strongest intensification, a high deviation of the repetition (32%) and high U value (264%), and a strongly positive matrix effect value (127%). A compound may have interfered with the m/z of δ-HCH. Generally, matrix effects were strongly positive in ß-HCH, δ-HCH, and methoxychlor in human plasma, in 4,4′-DDT, methoxychlor in chicken liver, and consistently negative in shearwater stomach oil (Table S3). With the re-quantification by external calibration of stomach oil quality controls, 2,4′-DDD showed realistic recovery and RSD of 47 ± 14%, but 4,4′-DDT had a very low recovery of 25 ± 30%. Some other OCPs (endrin, aldrin, ß-endosulfan, methoxychlor) and higher chlorinated PCBs were not recovered well (PCB 194) or had high RSD (PCB 180) in stomach oil and dibenz[a,h]anthracene had slightly higher and not fully satisfactory RSD, U, ME values. MDL were mostly below 1 ng/g or in the low ng/g range (Table 2) which compared to studies that used whole blood is a little higher (Dulsat-Masvidal et al. 2023; Campioni et al. 2024). MDL varies according to the matrix and extraction method used, for example, MDL for PAHs was higher in the raptor liver than in blood (Morin-Crini et al. 2022). MDL for POPs in seabird eggs was 1–1.12 ng/g (Elliott et al. 2023), and PAHs in loon plasma was 5 ng/g a similar range to ours (Paruk et al. 2016).

The complexity of the studied matrices favours deviations in the desired recovery and increased RSD for certain OCPs and PAHs. In biological matrices, lipids and other constituents such as salts, hormones, or proteins may generate interferences leading to signal enhancement or suppression which varies unpredictably with matrix type (Kim et al. 2016). Moradi et al. (2023) report differences in recovery and RSD between plasma and serum for acenaphthylene, anthracene, fluorene, and phenanthrene. Morin–Cringi et al. (2022) report higher RSD for phenanthrene, benzo(k)fluoranthene, and dibenz[a,h]anthracene in blood and fluoranthene, pyrene, and benzo[g,h,i]perylene in the liver. Good recoveries were achieved for PAHs in avian blood cells and plasma (60.0 to 92.4% for BCs and from 52.5 to 109% for plasma) using QuEChERS extraction followed by phospholipid solid-phase extraction clean-up and HPLC analysis although naphthalene, acenaphthylene, and fluoranthene were not available in the matrix spike samples of plasma (Provatas et al. 2015). The same study reported higher MDL in plasma than blood cells (4.14–12.4 ng/g for BCs and from 7.70 to 41.6 ng/g for plasma). Especially in the case of stomach oil, impurities hamper satisfactory recoveries as shown in our results. Zhao et al. (2013) analysed soybean oil applying a similar extraction/clean-up method (LLE with acetonitrile/acetone 60/40 v/v and 20 mL SPE cartridges) and HPLC analysis and reported similar recoveries (60–77%) for chrysene, benzo-(b)fluoranthene, benzo(a)perylene, benzo(k)fluoranthene, dibenz[a,h]anthracene, benzo[g,h,i]perylene, and indeno[1,2,3-cd]pyrene. A previous study on vegetable oils (Wu 2012) achieved good recoveries and repeatability when washing their samples twice with 50 mL of heated 4% saline solution. Zhang et al. (2017) achieved good recoveries for PAHs in vegetable oil by using magnetic dispersive solid-phase extraction, which also helped to minimize lipid impurities. Referring to the matrix effect levels mentioned by Li et al. (2016), most compounds in this study showed strong matrix effects above ± 50% in all matrices, which is expected for biological matrices (Oró-Nolla et al. 2023). Matrix-matched calibration may help to reduce the overestimation of the signal (Kim et al. 2016). However, the use of IS for correction would normally compensate for the matrix effects as we achieved satisfactory recoveries in most target compounds.

For acenaphthene (0.50–1.1 ng/mL), hexachlorobenzene (< 0.01–0.50 ng/mL), phenanthrene (1.10–2.00 ng/mL), fluoranthene (< 0.01–0.40 ng/mL), pyrene (0–1.00 ng/mL), and methoxychlor (0.30–4.30 ng/mL), low background contamination was detected in the procedural blank vials, and mean peak areas of the blanks were subtracted from the areas of the sample before quantification. Background levels of some POPs were detected in the matrix blanks of stomach oil, as pooled environmental samples were used (Table S5). In these cases, the contribution of detected compounds was subtracted before the recovery determination. Among matrices, the lowest uncertainty and matrix effects were observed in the liver, followed by plasma, and the highest in stomach oil (Table 2; Table S3). As the liver was the only matrix processed as a dry powder, it can be assumed that the water content of the sample may influence solvent accessibility, and extraction efficiency is improved. According to the analytical quality control and method validation procedures for food and feed proposed by the European Union (Pihlström et al. 2021), the U value should be below 50% to maintain interlaboratory standards. In this study, we obtained U below 50% for 54 (plasma), 67 (liver), and 41 (stomach oil) of our target compounds. The threshold of 50% has been established for food and feed not necessarily for complex biological matrices like the ones used in this study. Therefore, we believe that the reported values for U are acceptable.

### Pollutants detected in shearwaters

**Fig. 1:**
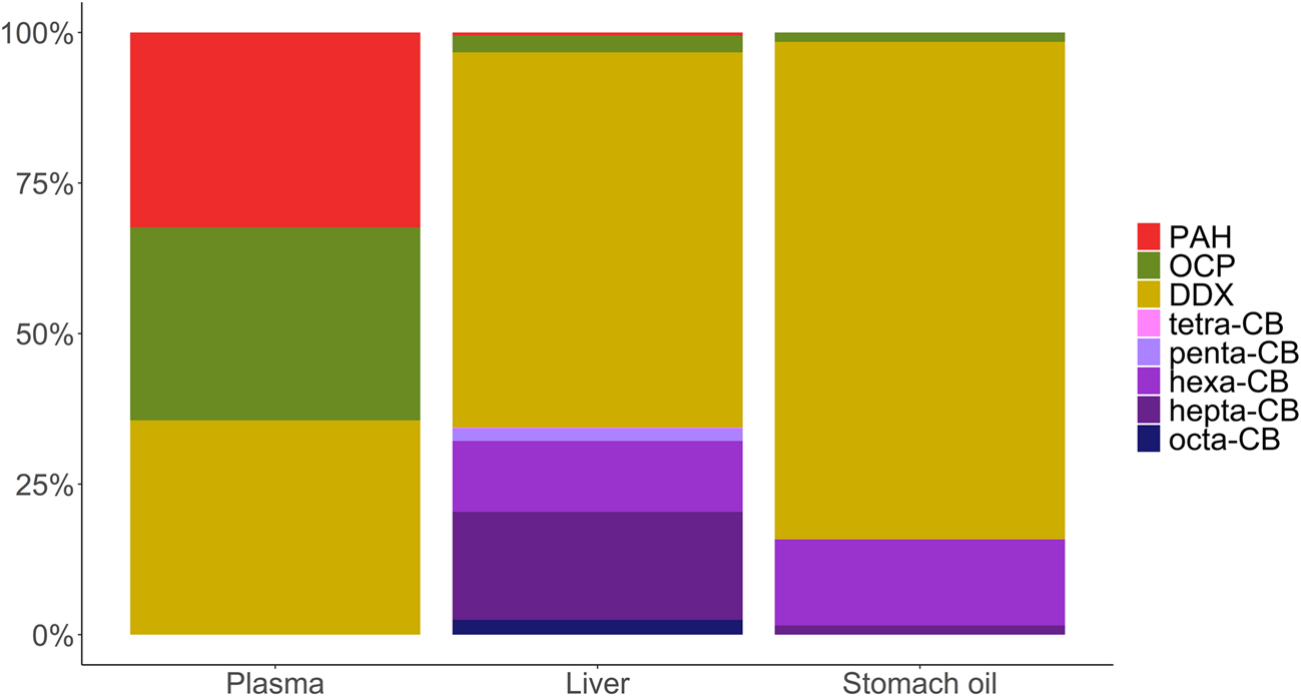
The percentage composition of all analysed pollutant classes in the environmental samples from Scopoli’s shearwaters (*Calonectris diomedea*). Plasma (*n*=21), liver (*n*=10), stomach oil (*n*=6). Only concentrations above MDL are included

**Fig. 2:**
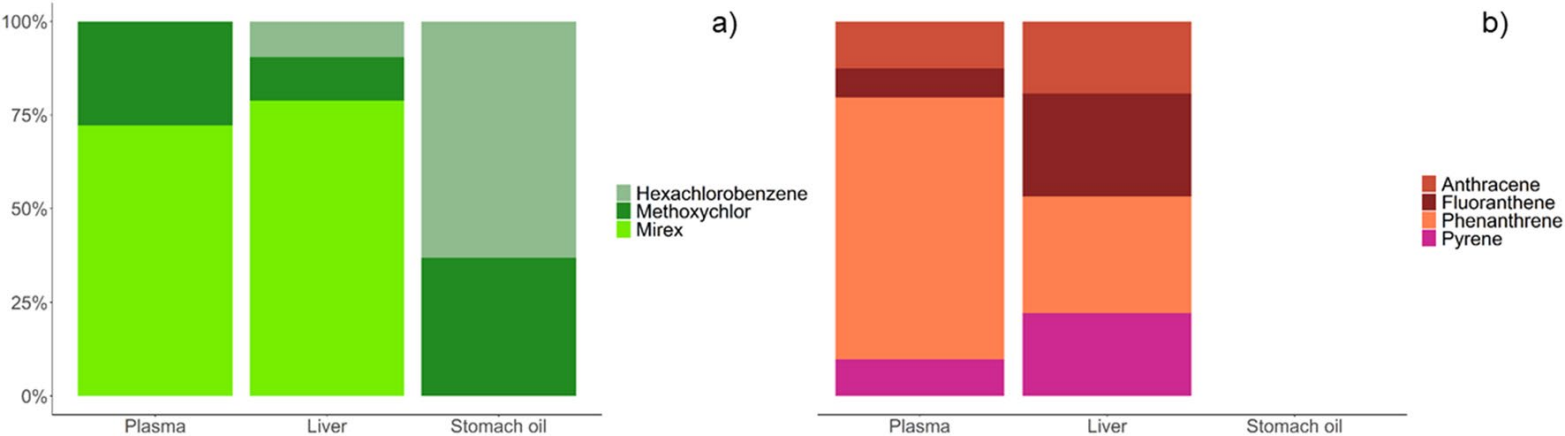
The percentage composition of **a**) PAHs and **b**) OCPs (excluding DDXs) in the environmental samples from Scopoli’s shearwaters (*Calonectris diomedea*). Plasma (*n*=21), liver (*n*=10), stomach oil (*n*=6). Only concentrations above MDL are included

In shearwaters, we detected concentrations above MDL for 28 out of our 68 targeted compounds. These consisted of four PAHs, seven OCPs, and 17 PCBs which are listed in Table 3. Among the three seabird matrices in this study, liver samples exhibited the highest pollutant concentrations, followed by stomach oil and plasma. The dominant compound in all three matrices was 4,4′-DDE (Table 3), detected in all liver and stomach oil samples and only in six out of 21 of the plasma samples. Generally, the concentrations deviated greatly from sample to sample. In the liver, there was one sample with higher concentrations compared to the others, resulting in high standard deviations (Table 3). This sample could be suitable for further retrospective non-targeted analyses. The composition and detection rate of POPs and PAHs varied among the matrices (Fig. 1), while in plasma, PCBs were mainly below the detection limit PAHs phenanthrene, fluoranthene, and pyrene were prevalent, and methoxychlor was detected in most of the samples. Plasma concentrations of 4,4′-DDE were lower but comparable to previously measured blood concentrations in this colony (Costantini et al. 2017). Interestingly, previous studies found higher concentrations of ∑PCBs than ∑DDT metabolites in the blood and liver of Linosan shearwaters (Renzoni et al. 1986; Costantini et al. 2017). Also in Bermuda petrels (Campioni et al. 2024) and giant petrels (Roscales et al. 2016), the blood concentrations of single indicator PCBs were similar or slightly higher than 4,4′-DDE. Even though the quality parameters for 4,4′-DDE are good, it cannot be excluded that some matrix effects that we faced in the quality controls of other DDT metabolites are causing the high concentrations. However, the concentration of 4,4′-DDE was high relatively in all three matrices. Commonly, 4,4′-DDE is the most prevalent of the DDT metabolites in wild birds, and this was the case in previous studies in the blood of other Mediterranean shearwaters (Roscales et al. 2010; Costantini et al. 2017), and temporal shifts of pollutant profiles are possible as Arctic eiders showed increasing trends of 4,4′-DDE blood concentrations while PCBs remained stable over a time period between 2007 and 2017 (Bustnes et al. 2024). The liver had a high prevalence and concentrations of PCB congeners (#153, #138, #187, #183, #128, #170, and #180), and the OCP mirex was detected in five out of the ten samples. Generally, the liver samples showed a dominance of higher chlorinated biphenyls, such as hexa-, hepta-, and octachlorinated biphenyls, compared to tetra- and pentachloro biphenyls (Fig. 1). The main contributor to OCPs after 4,4′-DDE in the liver was mirex (Fig. 2a). The proportion of PAHs in liver differed slightly from plasma, but phenanthrene contributed to the profile in both matrices (Fig. 2b). In stomach oil, PCB #138 was detected in five out of six samples, and methoxychlor was most prevalent after 4,4′-DDE. Interestingly, in stomach oil, we did not detect any PAHs (Figs. 1 and 2b).

**Table 3:** Mean ± standard deviation (SD), lower (LCI_95) and upper (UCI_95) 95% confidence intervals, minimum (min) and maximum (max) concentrations and percent of censoring (Cen) of detected compounds in native samples of plasma (*n* = 21), liver (*n* = 10) and stomach oil (*n* = 6) of Scopoli’s Shearwaters (*Calonectris diomedea*), expressed in ng/g w.w. Estimates of the shape and scale parameters (mean, SD and 95 confidence interval of the mean) for censored observations were calculated using the non-parametric Kaplan–Meier method from the R package EnvStats

	Plasma						Liver						Stomach Oil					
	Mean (ng/g w.w.) ± SD	LCI_95	UCI_95	Min	Max	Cen	Mean (ng/g w.w.) ± SD	LCI_95	UCI_95	Min	Max	Cen	Mean (ng/g w.w.) ± SD	LCI_95	UCI_95	Min	Max	Cen
Hexachlorobenzene	< MDL	NA	NA	0.00	1.53	100	4.66	NA	NA	3.08	4.66	90	2.96	NA	NA	2.38	2.96	83
Phenanthrene	2.23 ± 4.72	0.70	3.80	0.32	21.4	71	2.86 ± 2.81	1.36	4.36	0.95	9.71	40	< MDL	NA	NA	0.00	0.54	100
Anthracene	0.40± 0.03	NA	NA	0.39	0.54	90	1.77	NA	NA	1.28	1.77	90	< MDL	NA	NA	0.00	0.75	100
PCB 61	< MDL	NA	NA	0.00	0.84	100	3.08	NA	NA	0.00	3.08	90	< MDL	NA	NA	0.00	2.50	100
Fluoranthene	0.25 ± 0.12	0.21	0.29	0.22	0.76	81	2.53 ± 1.14	1.89	3.3	1.56	5.44	0	< MDL	NA	NA	0.00	0.46	100
PCB 99	< MDL	NA	NA	0.00	0.68	100	8.05 ± 11.7	1.74	14.3	3.13	42.8	60	< MDL	NA	NA	0.00	1.93	100
Pyrene	0.31 ± 0.12	0.27	0.35	0.22	0.65	52	2.03 ± 1.14	1.41	2.83	1.04	4.94	0	< MDL	NA	NA	0.00	0.40	100
4,4′-DDE	3.00 ± 5.21	1.60	4.40	0.41	14.9	71	1090 ± 2182	291	2513	115	7207	0	243 ± 136	138	397	123	507	0
PCB 85	< MDL	NA	NA	0.00	1.41	100	8.34	NA	NA	3.39	8.34	90	< MDL	NA	NA	0.00	2.94	100
PCB 110	< MDL	NA	NA	0.00	0.63	100	4.07	NA	NA	1.96	4.07	90	< MDL	NA	NA	0.00	1.59	100
PCB 149	< MDL	NA	NA	0.00	1.36	100	2.73	NA	NA	2.06	2.73	90	< MDL	NA	NA	0.00	2.29	100
2,4′-DDT	< MDL	NA	NA	0.00	0.44	100	1.72	NA	NA	1.59	1.72	90	1.22	NA	NA	0.93	1.22	83
PCB 146	< MDL	NA	NA	0.00	1.41	100	14.5 ± 19.5	3.72	25.3	2.53	66.7	40	< MDL	NA	NA	0.00	3.21	100
4,4′-DDD	< MDL	NA	NA	0.00	0.38	100	< MDL	NA	NA	0.00	1.61	100	1.62 ± 0.97	1.18	2.06	1.02	3.65	67
PCB 153	< MDL	NA	NA	0.00	4.10	100	86.6 ± 108	33.6	176	12.7	355	0	34.6 ± 8.30	26.7	44.1	24.3	47.1	0
PCB 118	< MDL	NA	NA	0.00	0.55	100	16.4	NA	NA	1.78	16.4	90	< MDL	NA	NA	0.00	2.23	100
4,4′-DDT*	0.51 ± 0.8	0.39	0.62	0.26	3.68	90	< MDL	NA	NA	1.91	1.91	100	< MDL	NA	NA	0.00	0.29	100
PCB 138	< MDL	NA	NA	0.00	1.08	100	48.5 ± 67.4	20.0	93.1	9.59	230	0	8.13 ± 4.70	3.78	15.5	2.03	15.7	17
PCB 187	< MDL	NA	NA	0.00	1.44	100	45.2 ± 68.8	3.54	86.9	6.44	244	20	< MDL	NA	NA	0.00	3.37	100
PCB 183	< MDL	NA	NA	0.00	1.56	100	92.1 ± 129	14.4	169	6.94	415	20	4.53 ± 0.90	4.28	4.77	3.90	6.00	67
PCB 128	< MDL	NA	NA	0.00	1.32	100	31.0 ± 51.9	3.57	58.5	2.22	171	50	< MDL	NA	NA	0.00	3.14	100
PCB 167	< MDL	NA	NA	0.00	1.08	100	4.39 ± 3.73	2.61	6.20	2.28	14.5	60	< MDL	NA	NA	0.00	3.52	100
PCB 156	< MDL	NA	NA	0.00	1.11	100	18.8 ± 24.2	9.78	27.7	2.31	79.6	60	< MDL	NA	NA	0.00	3.91	100
Methoxychlor*	0.88 ± 0.77	0.63	1.12	0.42	3.14	57	5.66	NA	NA	2.84	5.66	90	1.73 ± 3.28	0.00	4.25	0.05	9.05	33
PCB 180	< MDL	NA	NA	0.00	1.96	100	142 ± 146	59.6	283	21.0	447	0	< MDL	NA	NA	0.00	3.35	100
PCB 170	< MDL	NA	NA	0.00	1.72	100	34.0 ± 39.6	11.5	56.5	5.71	129	30	< MDL	NA	NA	0.00	3.64	100
Mirex	2.28	NA	NA	0.35	2.38	95	38.3 ± 46.4	19.1	57.6	1.74	119	50	< MDL	NA	NA	0.00	1.14	100
PCB 194	< MDL	NA	NA	0.00	1.82	100	43.4 ± 56.4	16.8	69.9	4.41	175	50	< MDL	NA	NA	0.00	3.61	100

^*^4,4′-DDT and methoxychlor showed signal intensification in liver quality controls

## Conclusion

This study presents a simple and direct method designed to detect POPs and PAHs in small volumes of three seabird matrices, which pose challenges to the analytical set-up due to their complex composition. We were able to recover 68 targeted compounds in spiked plasma and 67 compounds in liver and stomach oil. In few cases, recoveries were high due to matrix effects. Sufficiently good uncertainty values indicate the excellent performance of the extraction and analytical protocol. In adult and juvenile shearwaters from a remote Mediterranean colony, we detected 4 POPs (range 0.45–14.9 ng/g) and 4 PAHs (range 0.24–21.4 ng/g) in Plasma; 22 POPs (range 1.72–7207 ng/g) and 4 PAHs (range 1.04–9.71 ng/g) in liver; 8 POPs (range 0.17–507 ng/g), and no PAHs in stomach oil. This indicates bioaccumulation along all life stages and especially the exposure of young chicks through their first food source: stomach oil. Differences in pollutant patterns in the matrices were observed, which provides insight into specific bioaccumulation patterns related to exposure scenarios.

## Supplementary Information

Below is the link to the electronic supplementary material.

Supplementary file1 (PDF 683 KB)

## Data Availability

Data, associated metadata, and calculation tools not presented are available from the corresponding author (lucie.michel@bio.uni-giessen.de).
