# Supplementary material for: Analysis of organochlorines and polycyclic aromatic hydrocarbons designed for pollutant biomonitoring in three seabird matrices
Source: Environ Sci Pollut Res Int. 2024 Jul 9;33(22):11126–39. doi: 10.1007/s11356-024-34174-0 (PMC13415271; doi:10.1007/s11356-024-34174-0)
Supplement: Supplementary file 1 — Supplementary file1 (PDF 683 KB) [file 11356_2024_34174_MOESM1_ESM.pdf]

Supporting information

Title of the manuscript

Analysis of organochlorines and polycyclic aromatic hydrocarbons designed for pollutant biomonitoring in three seabird matrices

Journal name

Environmental Science and Pollution Research

Author names and affiliations

- Lucie Michel*, University of Giessen, Animal Ecology and Systematics, Giessen, Germany
  - E-mail: lucie.michel@bio.uni-giessen.de
- Bernat Oró-Nolla, IDAEA-CSIC, Department of Environmental Chemistry, Barcelona, Spain
- Giacomo Dell'Omo, Ornis Italica, Roma, Italy
- Petra Quillfeldt, University of Giessen, Animal Ecology and Systematics, Giessen, Germany
- Sílvia Lacorte, IDAEA-CSIC, Department of Environmental Chemistry, Barcelona, Spain

Table of content:

Table S1	Names, compound class, formulae, CAS-Numbers and supplier details of native and internal standards used in this study
Table S2	Additional quality parameters for 68 target POPs in elution order. Second and third diagnostic ions in m/z, slope, stable parameter (b) and R^2 for the calibration curve
Table S3	Matrix effects (ME) for each target compound.
Table S4	Summary table listing and categorizing issues that occurred in the spiked quality controls and how we resolved them using external calibration and explained them using uncertainty and matrix effect values.
Table S5	Compounds with peak areas and vial concentrations [ng/mL] in matrix blanks of pooled stomach oil, which were subtracted from the quality control samples

Table S1 Names, compound class, formulae, CAS-Numbers and supplier details of native and internal standards used in this study

**Target analytes**				
**Name**	**Compound class**	**Formula**	**CAS**	**Supplier and packaging unit**
2,4'-DDE	DDX	$C_{14}H_8Cl_4$	3424-82-6	AccuStandard, (New Haven, CT, USA) 10 ng/µL in Toluene
4,4'-DDE	DDX	$C_{14}H_8Cl_4$	72-55-9	
2,4'-DDD	DDX	$C_{14}H_{10}Cl_4$	53-19-0	
4,4'-DDD	DDX	$C_{14}H_{10}Cl_4$	72-54-8	
2,4'-DDT	DDX	$C_{14}H_9Cl_5$	789-02-6	
4,4'-DDT	DDX	$C_{14}H_9Cl_5$	50-29-3	
Aldrin	OCP	$C_{12}H_8Cl_6$	309-00-2	
α-HCH	OCP	$C_6H_6Cl_6$	319-84-6	
β-HCH	OCP	$C_6H_6Cl_6$	319-85-7	
δ-HCH	OCP	$C_6H_6Cl_6$	319-86-8	
γ-HCH	OCP	$C_6H_6Cl_6$	608-73-1	
Dieldrin	OCP	$C_{12}H_8Cl_6O$	60-57-1	
α -endosulfan	OCP	$C_9H_6Cl_6O_3S$	33213-65-9	
β-endosulfan	OCP	$C_9H_6Cl_6O_3S$	959-98-8	
Endrin	OCP	$C_{12}H_8Cl_6O$	72-20-8	
Heptachlor	OCP	$C_{10}H_5Cl_7$	76-44-8	
Hexachlorobenzene	OCP	C_6Cl_6	118-74-1	
Isodrin	OCP	$C_{12}H_8Cl_6$	465-73-6	
Methoxychlor	OCP	$C_{16}H_{15}Cl_3O_2$	72-43-5	
Mirex	OCP	$C_{10}Cl_{12}$	2385-85-5	
Oxychlordane	OCP	$C_{10}H_4Cl_8O$	26940-75-0	
PCB 16	PCB	$C_{12}H_7Cl_3$	38444-78-9	LGC Limited (Teddington, UK) 10 mg neat
PCB 24	PCB	$C_{12}H_7Cl_3$	55702-45-9	LGC Limited (Teddington, UK) 10 mg neat
PCB 38	PCB	$C_{12}H_7Cl_3$	53555-66-1	LGC Limited (Teddington, UK) 10 mg neat
PCB 49	PCB	$C_{12}H_6Cl_4$	41464-40-8	LGC Limited (Teddington, UK) 20 mg neat
PCB 52	PCB	$C_{12}H_6Cl_4$	35693-99-3	LGC Limited (Teddington, UK) 10 mg neat

PCB 61	PCB	$C_{12}H_6Cl_4$	33284-53-6	LGC Limited (Teddington, UK) 10 mg neat
PCB 62	PCB	$C_{12}H_6Cl_4$	54230-22-7	LGC Limited (Teddington, UK)
PCB 65	PCB	$C_{12}H_6Cl_4$	33284-54-7	LGC Limited (Teddington, UK)
PCB 77	PCB	$C_{12}H_6Cl_4$	32598-13-3	LGC Limited (Teddington, UK) 25 mg neat
PCB 85	PCB	$C_{12}H_5Cl_5$	65510-45-4	LGC Limited (Teddington, UK) 10 mg neat
PCB 99	PCB	$C_{12}H_5Cl_5$	38380-01-7	LGC Limited (Teddington, UK) 5 mg neat
PCB 101	PCB	$C_{12}H_5Cl_5$	37680-73-2	LGC Limited (Teddington, UK) 10 mg neat
PCB 110	PCB	$C_{12}H_5Cl_5$	38380-03-9	LGC Limited (Teddington, UK) 5 mg neat
PCB 116	PCB	$C_{12}H_5Cl_5$	18259-05-7	LGC Limited (Teddington, UK) 10 mg neat
PCB 118	PCB	$C_{12}H_5Cl_5$	31508-00-6	LGC Limited (Teddington, UK) 10 mg neat
PCB 128	PCB	$C_{12}H_4Cl_6$	38380-07-3	LGC Limited (Teddington, UK) 25 mg neat
PCB 138	PCB	$C_{12}H_4Cl_6$	35065-28-2	LGC Limited (Teddington, UK) 10 mg neat
PCB 146	PCB	$C_{12}H_4Cl_6$	51908-16-8	LGC Limited (Teddington, UK) 5 mg neat
PCB 149	PCB	$C_{12}H_4Cl_6$	38380-04-0	LGC Limited (Teddington, UK) 5 mg neat
PCB 153	PCB	$C_{12}H_4Cl_6$	35065-27-1	LGC Limited (Teddington, UK) 10 mg neat
PCB 156	PCB	$C_{12}H_4Cl_6$	38380-08-4	LGC Limited (Teddington, UK) 10 mg neat
PCB 167	PCB	$C_{12}H_4Cl_6$	52663-72-6	LGC Limited (Teddington, UK) 10 mg neat
PCB 170	PCB	$C_{12}H_3Cl_7$	35065-30-6	LGC Limited (Teddington, UK) 5 mg neat
PCB 180	PCB	$C_{12}H_3Cl_7$	35065-29-3	LGC Limited (Teddington, UK) 5 mg neat
PCB 183	PCB	$C_{12}H_3Cl_7$	52663-69-1	LGC Limited (Teddington, UK) 5 mg neat
PCB 189	PCB	$C_{12}H_3Cl_7$	39635-31-9	LGC Limited (Teddington, UK) 5 mg neat
PCB 194	PCB	$C_{12}H_2Cl_8$	35694-08-7	LGC Limited (Teddington, UK) 5 mg neat
Naphthalene	PAH	$C_{10}H_8$	91-20-3	

Acenaphthylene	PAH	$C_{12}H_8$	208-96-8	AccuStandard, (New Haven, CT, USA) 200 ng/µL in Toluene
Acenaphthene	PAH	$C_{12}H_{10}$	83-32-9	
Fluorene	PAH	$C_{13}H_{10}$	86-73-7	
Phenanthrene	PAH	$C_{14}H_{10}$	85-01-8	
Anthracene	PAH	$C_{14}H_{10}$	120-12-7	
Fluoranthene	PAH	$C_{16}H_{10}$	206-44-0	
Pyrene	PAH	$C_{16}H_{10}$	129-00-0	
1,2-benzanthracene	PAH	$C_{18}H_{12}$	56-55-3	
Chrysene	PAH	$C_{18}H_{12}$	218-01-9	
Benzo[b]fluoranthene	PAH	$C_{20}H_{12}$	205-99-2	
Benzo[k]fluoranthene	PAH	$C_{20}H_{12}$	207-08-9	
Benz[a]pyrene	PAH	$C_{20}H_{12}$	50-32-8	
Indeno[1,2,3-cd]pyrene	PAH	$C_{22}H_{12}$	193-39-5	
Dibenz[a,h]anthracene	PAH	$C_{22}H_{14}$	53-70-3	
Benzo[g,h,i]perylene	PAH	$C_{22}H_{12}$	191-24-2	
**Internal standards**				
Pentachlorobenzene $_{13}C^6$	OCP	C_6HCl_5	-	AccuStandard, (New Haven, CT, USA) 200 ng/µL in Toluene
Naphthalene D8	PAH	$C_{10}H_8$	1146-65-2	Sigma-Aldrich 4000 ng/µL
Acenapthene D10	PAH	$C_{12}H_{10}$	15067-26-2	
Phenanthrene D10	PAH	$C_{14}H_{10}$	1517-22-2	
Chrysene D12	PAH	$C_{18}H_{12}$	1719-03-5	
Perylene D12	PAH	$C_{20}H_{12}$	1520-96-3	
4,4'-DDE-D8	DDX	$C_{14}H_8Cl_4$	93952-19-3	LGC Limited (Teddington, UK)10 mg neat
PCB 73	PCB	$C_{12}H_6Cl_4$	74338-23-1	LGC Limited (Teddington, UK)10 mg neat
PCB 97	PCB	$C_{12}H_5Cl_5$	41464-51-1	LGC Limited (Teddington, UK) 10 mg neat
PCB 126	PCB	$C_{12}H_5Cl_5$	57465-28-8	LGC Limited (Teddington, UK) 10 mg neat
PCB 151	PCB	$C_{12}H_4Cl_6$	52663-63-5	LGC Limited (Teddington, UK) 5 mg neat
PCB 157	PCB	$C_{12}H_4Cl_6$	69782-90-7	LGC Limited (Teddington, UK) 10 mg neat
PCB 200	PCB	$C_{12}H_2Cl_8$	52663-73-7	LGC Limited (Teddington, UK) 5 mg neat

Table S2 Additional quality parameters for 68 target POPs in elution order. Second and third diagnostic ions in m/z, slope, stable parameter (b) and R^2 for the calibration curve

Compound	Ion 2 [m/z]	Ion 3 [m/z]	Slope	b	R^2
Naphthalene	129.0654	102.0464	0.89	-0.04	0.9985
Acenaphthene	154.0777	152.0620	0.81	-0.03	0.9974
Acenaphthylene	153.0654	126.0464	0.80	-0.22	0.9969
Fluorene	166.0777	163.0542	0.58	0.06	0.9982
α-HCH	218.9110	108.9606	0.66	-0.10	0.9993
Hexachlorobenzene	285.8066	281.8125	0.46	0.02	0.9975
β-HCH	182.9343	218.9110	1.49	-0.65	0.9937
δ-HCH	218.9110	108.9606	0.88	-0.27	0.9983
Phenanthrene	152.0620	179.0810	0.90	-0.04	0.9982
PCB 24	186.0230	257.9578	1.36	0.05	0.9990
Anthracene	176.0620	152.0620	0.84	-0.03	0.9985
PCB 16	220.9919	186.0230	0.42	0.01	0.9980
δ-HCH	182.9343	218.9110	0.65	-0.16	0.9986
Heptachlor	269.8125	100.0074	0.17	0.00	0.9991
PCB 52	291.9188	289.9218	1.21	-0.17	0.9907
PCB 49	219.98411	291.9188	0.84	0.04	0.9985
PCB 38	186.0230	257.9578	1.46	-0.08	0.9968
PCB 62	289.9218	219.9841	0.95	0.04	0.9980
PCB 65	289.9218	219.9841	0.94	0.10	0.9937
Aldrin	66.9990	263.4950	0.25	0.01	0.9987
Isodrin	194.9343	262.8564	0.25	-0.01	0.9970
PCB 61	289.9218	219.9841	1.09	0.06	0.9968
Heptachlorepoxide	354.8407	236.8407	0.15	0.00	0.9971
Oxychlordane	186.9107	115.0000	0.09	0.00	0.9986
Fluoranthene	200.0620	101.0385	0.95	-0.01	0.9976
2,4‘-DDE	247.9968	317.9345	8.86	-0.85	0.9951
trans-Chlordane	374.8224	376.8195	0.85	-0.14	0.9989
α-Endosulfan	259.9841	169.9684	0.49	-0.11	0.9979
cis-Chlordane	376.8195	374.8224	0.48	-0.05	0.9983
PCB 101	253.9451	327.8798	1.42	0.06	0.9986
PCB 99	253.9451	327.8798	2.00	0.10	0.9951

Pyrene	200.0620	201.0698	0.87	0.05	0.9967
PCB 116	253.9451	327.8798	1.47	0.09	0.9969
4,4'-DDE	247.9968	317.9345	4.46	0.20	0.9982
PCB 85	253.9451	323.9870	1.04	0.04	0.9975
PCB 110	327.8769	323.9870	2.34	0.09	0.9933
Dieldrin	79.0450	81.0334	0.26	-0.01	0.9983
2,4'-DDD	237.0046	165.0698	9.87	-0.04	0.9989
PCB 77	289.9218	219.9841	2.93	0.12	0.9988
PCB 149	361.9789	289.9032	1.62	0.07	0.9971
Endrin	242.9529	280.9266	0.23	-0.01	0.9986
β-Endosulfan	169.9684	192.9373	0.22	-0.02	0.9995
2,4'-DDT	237.0046	165.0698	10.1	-0.09	0.9975
PCB 146	361.9789	289.9032	0.65	0.04	0.9964
4,4'-DDD	165.0698	199.0309	3.32	-0.05	0.9998
PCB 153	361.9789	144.9032	0.83	0.02	0.9935
PCB 118	327.8769	323.9870	1.82	-0.15	0.9956
4,4'-DDT	165.0698	245.9997	0.93	-0.03	0.9962
PCB 138	361.9789	289.9032	0.72	0.13	0.9959
PCB 187	395.7989	324.0000	0.45	0.03	0.9927
PCB 183	395.7989	324.0000	0.50	0.02	0.9960
PCB 128	361.9789	144.9032	0.76	-0.03	0.9978
PCB 167	361.9789	289.9032	0.82	0.05	0.9969
PCB 156	361.9789	289.9032	1.17	0.05	0.9975
Methoxychlor	228.1101	152.0620	0.50	-0.02	0.9964
1,2-Benzanthracene	101.0385	226.0304	0.88	0.02	0.9976
Chrysene	226.0777	229.0967	0.81	0.02	0.9982
PCB 180	395.7989	397.8976	0.45	0.03	0.9972
PCB 170	395.7989	324.0000	0.77	0.01	0.9978
Mirex	236.8407	269.8125	0.70	-0.01	0.9998
PCB 189	395.7989	324.0000	0.87	0.05	0.9938
PCB 194	427.7629	431.0320	0.49	0.09	0.9931
Benzo[b]fluoranthene	250.0777	253.0967	4.47	-0.31	0.9961
Benzo[k]fluoranthene	250.0777	253.0967	4.91	-15.0	0.9995
Benz[a]pyrene	250.0777	253.0967	3.99	-10.8	0.9989

Indeno[1,2,3-cd]pyrene	274.0777	277.0967	1.86	-5.71	0.9972
Dibenz[a,h]anthracene	276.0933	274.0777	1.58	-4.16	0.9976
Benzo[g,h,i]perylene	274.0777	277.0967	1.78	-4.56	0.9973

Table S3 Matrix effects (ME) for each target compound

**Compound**	**Plasma ME [%]**	**Liver ME [%]**	**Stomach oil ME [%]**
Naphthalene	-45	-32	-19
Acenaphthene	-58	-65	-53
Acenaphthylene	-56	-60	-75
Fluorene	-53	-33	-43
α-HCH	-61	-60	-54
Hexachlorobenzene	-70	-68	-47
β-HCH	2	-64	-77
γ-HCH	-5	-54	-30
Phenanthrene	-44	-20	-38
PCB 24	-58	-40	-78
Anthracene	-49	-30	-54
PCB 16	-47	-29	-63
Heptachlor	-55	-93	-63
δ-HCH	127	-55	-57
PCB 52	-61	-34	-62
PCB 49	-63	-34	-63
PCB 38	-61	-26	-61
PCB 62	-63	-35	-67
PCB 65	-66	-38	-66
Aldrin	-68	-36	-92
Isodrin	-65	-36	-74
PCB 61	-70	-32	-65
Heptachlorepoxide	-49	-44	-76
Oxychlordane	-51	-43	-85
Fluoranthene	-50	-57	-56
2,4‘-DDE	-63	-67	-78
trans-Chlordane	-53	-68	-73
α-Endosulfan	-49	-61	-72
PCB 101	-54	-66	-76
PCB 99	-64	-62	-83
cis-Chlordane	-69	-58	-73
Pyrene	-48	-42	-63
PCB 116	-72	-65	-77
4,4’-DDE	-69	-60	-48
PCB 85	-69	-63	-76
PCB 110	-66	-61	-76
Dieldrin	-54	-64	-78
2,4‘-DDD	-43	-49	-59
PCB 77	-64	-75	-82

PCB 149	-71	-67	-75
Endrin	-61	-79	-88
β-Endosulfan	-2	-54	-94
2,4‘-DDT	-64	-58	-75
PCB 146	-72	-49	-68
4,4’-DDD	-64	-57	-69
PCB 153	-73	-50	-66
PCB 118	-65	-42	-67
4,4’-DDT	-11	101	-56
PCB 138	-75	-60	-67
PCB 187	-72	-35	-65
PCB 183	-71	-36	-79
PCB 128	-70	-53	-73
PCB 167	-71	-38	-65
PCB 156	-71	-50	-69
Methoxychlor	18	87	-56
1,2-Benzanthracene	-42	-29	-67
Chrysene	-40	-38	-52
PCB 180	-69	-52	-59
PCB 170	-66	-52	-78
Mirex	-71	-51	-78
PCB 189	-72	-51	-84
PCB 194	-80	-57	-86
Benzo[b]fluoranthene	-64	-60	-81
Benzo[k]fluoranthene	-52	-51	-76
Benz[a]pyrene	-57	-54	-80
Indeno[1,2,3-cd]pyrene	-59	-56	-86
Dibenz[a,h]anthracene	-61	-58	-91
Benzo[g,h,i]perylene	-58	-43	-91

Table S4 Summary table listing and categorizing issues that occurred in the spiked quality controls and how we resolved them using external calibration and explained them using uncertainty and matrix effect values.

Target compound	issue in quality control repetitions	signal intensified?	requantified with external calibration	signal remained intensified after ext cal?	Uncertainty	Matrix effects
**ß-HCH**	high RSD in stomach oil qc repetitions	yes, in plasma	yes	no	< 50 in plasma and stomach oil	small positive Matrix effect in plasma (2)
**γ-HCH**	high RSD in plasma, liver and stomch oil qc repetitions	yes, in plasma	yes	no	< 50 in plasma and stomach oil	very low negative effect in plasma (-5)
**δ-HCH**	high RSD in plasma qc repetitions	yes, in plasma	yes	yes, in plasma	high uncertainty in plasma 264	high positive matrix effect in plasma (127)
**Aldrin**	high RSD in stomach oil qc repetitions	no	no	no	< 50 in plasma and stomach oil	strong negative effect in stomach oil (-92)
**2,4'-DDD**		yes, in liver and stomach oil	yes	no	< 50 in plasma and stomach oil	negative effects in all matrices
**Endrin**	high RSD in liver and stomach oil qc repetitions	no	no	no	< 50 in liver and stomach oil	negative effect in stomach oil (-88)
**ß-endosulfan**	high RSD in stomach oil qc repetitions	yes, in plasma	yes	no	< 50 in plasma and stomach oil	very low negative effect in plasma (-2), strong negative effect in stomach oil (-94)
**4,4'-DDT**	high RSD in stomach oil qc repetitions	yes, in plasma and liver and stomach oil	yes	yes, in liver	< 50 in plasma and stomach oil	strong positive effect in liver (101)
**Methoxychlor**	high RSD in stomach oil qc repetitions	yes, in plasma and liver	yes	yes, in liver	< 50 in plasma and stomach oil	positive effect in plasma (18) and strong positive effect in liver (87)
**PCB 180**	high RSD in stomach oil qc repetitions	no	no	no	< 50 in stomach oil	negative effects in all matrices
**PCB 194**	high RSD in stomach oil qc repetitions	no	no	no	< 50 in plasma and stomach oil	negative effects in all matrices
**Benzo(g,h,i)perylene**	high RSD in stomach oil qc repetitions	no	no	no	< 50 in stomach oil	strong negative effect in stomach oil (-91)

Table S5 Compounds with peak areas and vial concentrations [ng/mL] in matrix blanks of pooled stomach oil, which were subtracted from the quality control samples

**Compound**	**peak area**	**concentration in ng/mL in vial**
Hexachlorobenzene	709108	2.2
PCB 99	99574	0.2
4,4'-DDE	8632109	5.8
PCB 149	165210	0.7
2,4'_DDT	402218	0.2
PCB 146	113739	0.5
PCB 153	1873288	7.4
PCB 118	646218	1.2
4,4'-DDT	118220	0.5
PCB 138	1478138	5.7
PCB 187	185708	1.1
PCB 180	671862	5.2
PCB 170	34334	0.3
PCB 189	19575	0.1
